# Preoperative hypofractionated radiotherapy for soft tissue sarcomas: a systematic review

**DOI:** 10.1186/s13014-022-02072-9

**Published:** 2022-09-14

**Authors:** Siyer Roohani, Felix Ehret, Marta Kobus, Anne Flörcken, Sven Märdian, Jana Käthe Striefler, Daniel Rau, Robert Öllinger, Armin Jarosch, Volker Budach, David Kaul

**Affiliations:** 1 Department of Radiation Oncology, Charité – Universitätsmedizin Berlin, corporate member of Freie Universität Berlin and Humboldt Universität zu Berlin, Augustenburger Platz 1, 13353 Berlin, Germany; 2 Berlin Institute of Health at Charité – Universitätsmedizin Berlin, Charitéplatz 1, 10117 Berlin, Germany; 3 Department of Hematology, Oncology and Tumor Immunology, Charité – Universitätsmedizin Berlin, corporate member of Freie Universität Berlin and Humboldt Universität zu Berlin, Augustenburger Platz 1, 13353 Berlin, Germany; 4 Centre for Musculoskeletal Surgery, Charité – Universitätsmedizin Berlin, corporate member of Freie Universität Berlin and Humboldt Universität zu Berlin, Campus Virchow Klinikum, Augustenburger Platz 1, 13353 Berlin, Germany; 5 Department of Surgery, Charité – Universitätsmedizin Berlin, corporate member of Freie Universität Berlin and Humboldt Universität zu Berlin, Berlin, Germany; 6 Institute of Pathology, Charité – Universitätsmedizin Berlin, corporate member of Freie Universität Berlin and Humboldt Universität zu Berlin, Berlin, Germany; 7 Charité – Universitätsmedizin Berlin, Berlin, Germany; German Cancer Consortium (DKTK), partner site Berlin, and German Cancer Research Center (DKFZ), 69120 Heidelberg, Germany

**Keywords:** Soft tissue sarcoma, Sarcoma, Radiotherapy, Hypofractionation, Preoperative radiotherapy, Neoadjuvant radiotherapy, Toxicity, Wound complications

## Abstract

**Background:**

Soft tissue sarcomas (STS) represent a diverse group of rare malignant tumors. Currently, five to six weeks of preoperative radiotherapy (RT) combined with surgery constitute the mainstay of therapy for localized high-grade sarcomas (G2–G3). Growing evidence suggests that shortening preoperative RT courses by hypofractionation neither increases toxicity rates nor impairs oncological outcomes. Instead, shortening RT courses may improve therapy adherence, raise cost-effectiveness, and provide more treatment opportunities for a wider range of patients. Presumed higher rates of adverse effects and worse outcomes are concerns about hypofractionated RT (HFRT) for STS. This systematic review summarizes the current evidence on preoperative HFRT for the treatment of STS and discusses toxicity and oncological outcomes compared to normofractionated RT.

**Methods:**

We conducted a systematic review of clinical trials describing outcomes for preoperative HFRT in the management of STS using PubMed, the Cochrane library, the Cochrane Central Register of Controlled Trials, ClinicalTrials.gov, Embase, and Ovid Medline. We followed the 2020 Preferred Reporting Items for Systematic Reviews and Meta-Analyses (PRISMA) guidelines. Trials on retroperitoneal sarcomas, postoperative RT, and hyperthermia were excluded. Articles published until November 30th, 2021, were included.

**Results:**

Initial search yielded 94 articles. After removal of duplicate and ineligible articles, 13 articles qualified for analysis. Eight phase II trials and five retrospective analyses were reviewed. Most trials applied 5 × 5 Gy preoperatively in patients with high-grade STS. HFRT courses did not show increased rates of adverse events compared to historical trials of normofractionated RT. Toxicity rates were mostly comparable or lower than in trials of normofractionated RT. Moreover, HFRT achieved comparable local control rates with shorter duration of therapy. Currently, more than 15 prospective studies on HFRT + / − chemotherapy are ongoing.

**Conclusions:**

Retrospective data and phase II trials suggest preoperative HFRT to be a reasonable treatment modality for STS. Oncological outcomes and toxicity profiles were favorable. To date, our knowledge is mostly derived from phase II data. No randomized phase III trial comparing normofractionated and HFRT in STS has been published yet. Multiple ongoing phase II trials applying HFRT to investigate acute and late toxicity will hopefully bring forth valuable findings.

**Supplementary Information:**

The online version contains supplementary material available at 10.1186/s13014-022-02072-9.

## Introduction

Soft tissue sarcomas (STS) are a heterogenous group of malignant tumors arising from mesenchymal tissue in virtually all anatomic locations and age groups [1, 2]. STS account for less than 1% of all tumor entities in adults and 7% in pediatric patients [3, 4]. The estimated incidence rate in Europe is 4–5 per 100 000 per year [5]. The World Health Organization applies two standard histopathological grading systems for STS based on histological, morphological and molecular characteristics [6–8]. This review will analyze data on adult patients with STS of the extremities and trunk and exclude retroperitoneal STS and trials on hyperthermia, which are discussed elsewhere [9, 10].

Owing to STS heterogeneity, the disease-associated morbidity and mortality are highly variable. Positive surgical margins, recurrent disease at presentation, histological grade, tumor depth, and previous local recurrences (LR) are independent risk factors for subsequent recurrences and mortality [11–14]. Moreover, specific histological subtypes, e.g., malignant peripheral nerve sheath tumors or myxofibrosarcomas, are associated with unfavorable clinical outcomes [11, 12, 15, 16]. In high-grade STS (G2-G3), current standard of care comprises surgery combined with preoperative conventionally fractionated RT, preferably carried out in sarcoma reference centers [17–19]. Preoperative (neoadjuvant) conventionally fractionated RT is applied over five to six weeks in daily fractions of 1.8–2.0 Gy to a total dose of 50–50.4 Gy [18, 20]. The role of perioperative chemotherapy remains controversial and depends on the above-mentioned risk factors [21]. Although preoperative RT causes higher wound complication rates, postoperative RT leads to irreversible fibrosis-related toxicities adversely affecting patients’ function. This has caused an increasing notion of preferring pre- over postoperative RT among radiation oncologists [22–26].

In daily practice, single doses higher than 2.2 Gy are usually considered as hypofractionated radiotherapy (HFRT), although no exact definition exists. It has been hypothesized that increasing radiation doses per fraction would raise the toxicity rate in normal tissue [27, 28]. Therefore, HFRT was mainly applied in palliative settings where fast symptom relief (e.g., pain relief in bone metastases) and lower total doses than in definitive RT settings are required. However, within the last two decades, further evidence on the efficacy and safety of hypofractionated therapy regimens has come from RT trials of breast cancer, prostate cancer, and rectal cancer, where hypofractionation is now routinely applied [29–31].

When comparing outcomes of different clinical trials, it is essential to bear in mind that over the last decades, RT has been—and is to this date—subject to tremendous technological advances. Technical innovation in all sections of radiation oncology (imaging, treatment planning, linear accelerators) have remarkably improved radiation precision and tolerability [32–34]. In line with this, a more recent trial applying modern radiation techniques and image guidance has shed new light on RT in STS: By using advanced and more precise radiation techniques, the investigators were able to reduce toxicity rates in preoperative, normofractionated RT for STS (10.5% of at least one grade ≥ 2 toxicity at two years vs. 35% in the SR-2 trial) [35].

Another rationale in favor of hypofractionation is based on radiobiological observations in STS. STS like liposarcomas and rhabdomyosarcomas are likely to have lower α/β ratios (< 10), making them rather sensitive to larger fraction sizes [36–38]. Rather interestingly, other tumor entities with similar α/β ratios of less than 10 (e.g., breast and rectal cancer) have shown similar local control (LC) rates after HFRT as compared to conventionally fractionated RT [39, 40].

Supporters of HFRT also argue with practical advantages of this therapy regimen. The treatment of STS at specialized, multidisciplinary sarcoma centers has shown beneficial outcomes for patients and improves overall survival (OS) [19, 41–43]. By shortening RT courses through hypofractionation without compromising patient outcomes, access to high-volume sarcoma centers can be particularly improved for immobile, frail, and elderly patients [44]. Shortening RT regimens is not only preferred by patients; it also reduces the economic burden on the health care system while increasing patient throughput at high-volume centers [45–49]. Especially during the COVID-19 pandemic, when medical care is less widely available, and patient contact is aimed to be reduced to a minimum, hypofractionation may constitute a preferred treatment modality [50].

To the best of our knowledge, no review has systematically analyzed the literature on preoperative HFRT regimens for STS treatment. To address this topic and give deeper insights into the advantages and drawbacks of hypofractionation, we conducted a systematic review of the literature to assess patient outcome parameters, toxicity rates, and feasibility. The current evidence and findings for preoperative HFRT in the treatment of STS in adults are summarized herein.

## Materials and methods

A systematic review of the literature was performed in accordance to the guidelines of the 2020 Preferred Reporting Items for Systematic Reviews and Meta-Analyses (PRISMA, PRISMA 2020 study protocol checklist, Additional file 1: The PRISMA 2020 checklist, supplementary materials) [51]. The databases PubMed, ClinicalTrials.gov, the Cochrane library and the Cochrane Central Register of Controlled Trials, Embase, and Ovid Medline were used. Variably combined search items included “hypofractionation”, “soft tissue sarcoma”, “radiotherapy”, “trunk and extremity sarcoma”, “neoadjuvant radiotherapy”, “oncological outcomes”, “wound complication”, “toxicity”, “safety”, “feasibility” and “efficacy”. For ongoing clinical trials, the ClinicalTrials.gov webpage was used with the following search items: “soft tissue sarcoma”, “hypofractionated radiotherapy” and “radiotherapy”. Databases were searched on November 30th, 2021 (Table 1). No filters or limits were applied. All English studies published before November 30th, 2021, were included. The first reviewer (S.R.) excluded duplicates, trials on hyperthermia or postoperative RT (trials adding postoperative boost to preoperative RT were not excluded), trials not matching the search items and trials on retroperitoneal sarcomas (due to their profound differences regarding the clinical course, treatment, and histological subtypes). The following types of articles were included: randomized controlled trials, open-label trials, retrospective analyses, phase II and III clinical trials, as well as single and multicenter trials applying preoperative HFRT on adults (≥ 18 years) with STS. This review was not registered.

**Table 1:** Inclusion and exclusion criteria

Category	Inclusion criteria	Exclusion criteria
Study design	Any except narrative reviews and systematic reviews	Systematic reviews Narrative reviews
Population	Age: ≥ 18 years	Pediatric patients (< 18 years)
	Sex: Any	Retroperitoneal sarcoma
	Race: Any	
	Disease: Soft tissue sarcomas located at the extremities and/or trunk	Other location than extremity or trunk
	Histological grade: Any	
	Stage: Localized	
Intervention	Hypofractionated RT (> 2.2 Gy/fraction/day) Neoadjuvant RT Neoadjuvant and/or adjuvant chemotherapy Surgical resection	Normofractionated RT (1.8–2.2 Gy/fraction) Hyperfractionated RT (< 1.8 Gy/fraction) Hyperthermia Postoperative RT (trials adding postoperative boost to preoperative RT were not excluded)
Outcomes	Acute toxicity including wound complications Late toxicity OS DFS LC LR LRFS	
Date range	Until November 30th, 2021	

*DFS* disease-free survival, *LC* local control, *LR* local recurrence, *LRFS* local recurrence-free survival, *OS* overall survival, *RT* radiotherapy

### Data items

The data items extracted from all eligible studies were author list, publication date, number of patients, patient demographics, histological subtypes of STS, anatomical locations, median tumor size, dose per fraction, number of fractions, time from RT to surgery and from surgery to RT, chemotherapy regimens, median follow-up, overall survival, local control, local recurrence, local recurrence-free survival (LRFS), progression-free survival (PFS), disease-free survival (DFS), wound complication (WC)- and late toxicity rates. If an article lacked any data on the aforementioned items, the specific field was left blank in the summary table resulting in lower validity and comparability of the respective trial. After initial selection of data items by the first reviewer (S.R.), the second reviewer (D.K.) checked for suitability and accuracy.

### Quality control and assessment

To ensure adequate quality standards for included articles, both the titles, abstracts, and full texts were thoroughly examined by the first reviewer. All resources obtained online were saved as PDF files in case the online record was edited or removed. Risk of bias was assessed individually for every study by using the Risk of Bias In Non-randomized Studies of Interventions tool (ROBINS-I) developed by the Cochrane Bias Methods Group [52] (Additional file 2: Risk of bias assessment according to ROBINS-I, Table 1). After initial evaluation by the first reviewer, the second reviewer then critically edited the bias assessment, list of results, data and added further articles, if required. In cases of uncertainty, the third reviewer (F.E.) gave critical input.

## Results

**Fig. 1:**
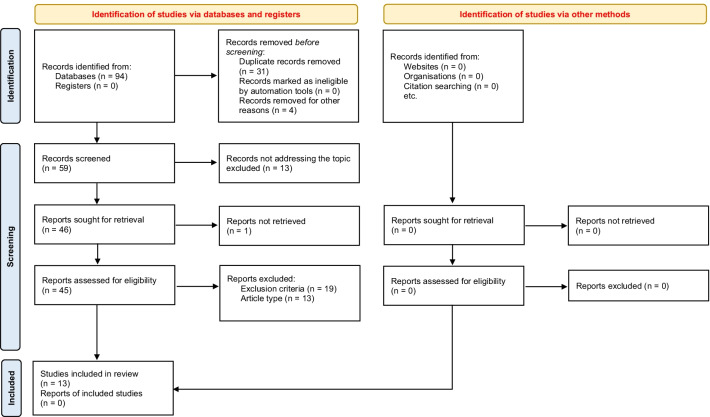
Flow diagram for study selection according to the PRISMA 2020 guidelines [51]

The PRISMA flow diagram depicted in Fig. 1 shows all initial search results, excluded articles and the final number of articles meeting the inclusion criteria. Systemically reviewed studies on preoperative hypofractionated radiotherapy are summarized in Table 2; major studies on conventionally fractionated radiotherapy are summarized in Table 3.

**Table 2:** Results. The table summarizes the current literature on preoperative hypofractionated RT for STS

Author	Year and country	Type of trial and inclusion criteria	N	Median age (years)	Sex ratio (♀:♂ in %)	Histologic grade	Location	Median tumor diameter	Fraction and dose; target Volume	EQD2/BEDα/β of 4 (Gy)
Koseła-Paterczyk et al. [53]	2021 Poland	Phase II single center trial Localized G2-G3 STS or G1 if >10cm	311	57	52 : 48	G1-2: 9.7% G3: 84.1% Unknown: 6.2%	LE: 72% UE: 16.7% Trunk: 11.3%	10 cm	5 x 5 Gy = 25 Gy CTV = GTV + 2cm transv.; + 4cm long. PTV = CTV + 0.7-1cm	37.5 Gy/ 56.3 Gy
Spalek et al. [54]	2021 Poland	Phase II single center trial Localized, marginally resectable G2-G3 STS	46	58	37 : 63	G2: 34.8% G3: 65.2%	LE: 63% UE: 15% Trunk: 22%	17.4 cm	5 x 5 Gy = 25 Gy CTV = GTV + 2cm transv.; + 4cm long. PTV = CTV + 0.7-1cm	37.5 Gy/ 56.3 Gy
Leite et al. [55]	2021 Brazil	Phase II single center trial Localized, extremity G2-G3 STS > 10 cm	25	42	44 : 56	G1-2: 21.7% G3: 78.3 %	LE: 60% UE: 40%	14 cm Pre-SBRT 10.5 cm Post-SBRT	5 x 8 Gy = 40 Gy CTV = GTV + 0.3-0.5 cm radial; + 2-3 cm long. PTV = CTV + 0.3cm	80 Gy/120 Gy
Potkrajcic et al. [56]	2021 Germany	Retrosp. Analysis Age >75 yrs., G2-G3 STS, localized on extremity/trunk	18	83.7	N/A	G2: 33.3% G3: 55.6% G2-3: 5.6% Unknown: 5.6%	LE: 55.6% UE: 27.8% Trunk: 16.6%	7.9 cm	5 x 5 Gy = 25 Gy CTV = GTV + 1.5cm radial; + 3cm long. PTV = CTV + 0.5-1 cm	37.5 Gy/ 56.3 Gy
Silva et al. [57]	2021 Brazil	Phase II single center trial Age 18-75, localized STS, not amenable to resection	18	53.5	56 : 44	G2: 11% G3: 89%	LE: 67% UE: 33%	8.9 cm	5 x 5 Gy = 25 Gy CTV = GTV + 1.5cm radial; + 4cm long. PTV = CTV + 1cm	37.5 Gy/ 56.3 Gy

RT modality	CTX	Time to surgery	Median FU (mths)	OS	LR	LC	LRFS	DFS	Acute toxicity	Late toxicity
3D-CRT: 95.8% IMRT: 3.9% VMAT: 0.3 % IGRT: 100%	30.2 % pre-OP AI or doxo/DTIC	2-4 days	57.3	63% 5 yrs	13.8% 5 yrs	N/A	N/A	46% 5 yrs.	Major WC: 24%:	Overall: 8.6%
3D-CRT: 6.5% VMAT: 41.3% IMRT: 52.2% IGRT: 100%	AI 3 cycles	6-8 weeks	24.4	53% 3 yrs	3 of 41 resected tumors (7%)	N/A	67% 2 yrs	N/A	Major WC: 34%. Overall: °1-°4: 44%	pending
SBRT IMRT VMAT IGRT	20% pre-op	8.6 weeks (median)	20.7	≈ 85% 3 yrs	0% 2 yrs	N/A	≈ 85% 3 yrs	N/A	Major WC: 28% 2°: Dermatitis: 48% Edema: 8% 3° Dermatitis:20%	1°: Fibrosis: 34.7% Edema: 21.7% 2°: Fibrosis: 4.3% Edema: 4.3% Stiffness: 13%
3D-CRT > IMRT/ VMAT	No	4.1 weeks (median)	5.1	100%	2 of 17 followed pts (11.8%)	92% 6 mths	N/A	84% 6 mths	Major WC: 29%	N/A
3D-CRT IMRT	AI 3 cycles	6 weeks (median)	29	95%	N/A	95%	N/A	72%	Major WC: 33%	1°: Fibrosis: 50% Stiffness: 16% Edema: 11% 2°: Fibrosis: 6% Stiffness: 6% Edema: 11%

Author	Year and country	Type of trial and Inclusion criteria	N	Median age (years)	Sex ratio (♀:♂ in %)	Histologic grade	Location	Median tumor diameter	Fraction and dose; target volume	EQD2/BEDα/β of 4 (Gy)
Koseła-Paterczyk et al. [58]	2020 Poland	Phase II single center trial Localized extremity or trunk MLPS, ≥ 5cm	27	43	48 : 52	Myxoid liposarcoma only G1-G2: 66.6% G3: 33.3%	LE only	13 cm	5 x 5 Gy = 25 Gy CTV = GTV + 2cm transv.; +4cm long. PTV = CTV + 0.7-1cm	37.5 Gy/ 56.3 Gy
Kalbasi et al. [47]	2020 USA	Phase II single center trial Localized extremity or trunk STS	50	<50: 28% 50-64: 22% 65-79: 40% >79: 10%	44 : 56	G1: 2 % G2: 38 % G3: 60 %	LE: 68% UE: 18% Trunk: 14%	26% ≤5 cm 50% >5 - ≤10 cm 24% >10 cm:	5 x 6 Gy = 30 Gy CTV = GTV + 1.5cm transv.; + 3cm long. PTV = CTV + 0.5cm	50 Gy/75 Gy
Parsai et al. [59]	2020 USA	Retrosp. Analysis Localized extremity or trunk STS	16	64	44 : 56	G2: 50% G3: 18.8% Unknown: 31.2%	LE: 62.5% UE: 25% Trunk: 12.5%	18.8% ≤5 cm 56.2% >5 - ≤10cm 18.8% >10 cm - ≤15 cm 6.2% >15 cm	n=1: 5 x 5 Gy = 25 Gy n=14: 5 x 6 Gy = 30 Gy n=1 5 x 8 Gy = 40 Gy Target volumes: according to RTOG-0630 [35]	n=1 37.5 Gy/ 56.3 Gy n=14 50 Gy/75 Gy n=1 80 Gy/120 Gy
Pennington et al. [60]	2018 USA	Retrosp. Analysis Localized, non-recurrent STS	116	46	40 : 60	G1: 0.9% G2: 13% G3: 79% Unknown: 7%	LE: 79% UE: 21%	17% ≤5cm 35% >5 - ≤10 cm 47% >10 cm	8 x 3.5 Gy = 28 Gy CTV = GTV + 4-5 cm long. PTV: N/A	35 Gy/52.5 Gy

RT modality	CTX	Time to surgery	Median FU (mths)	OS	LR	LC	LRFS	DFS	Acute toxicity	Late toxicity
3D-CRT > IMRT > VMAT	No	7 weeks (median)	27.1	25 of 27 pts (93%)	0%	N/A	N/A	100% 3 yrs G1-G2 50% 3 yrs G3	Wound dehiscence: 10.3% Wound infection: 17.2% Dermatitis: 1°: 34.4% 2°: 3.4% 3°: 3.4%	13.8 % Edema: 1°: 3.4% 2°: 3.4% Fibrosis °1: 3.4% °2: 3.4%
3D-CRT 20 IMRT 76% IGRT 96% Electron 4%	No	4 weeks (median)	29	84%	2 of 35 pts (5.7%)	N/A	N/A	N/A	Major WC: 32% Most common in LE °2 dermatitis: 8%	1°: Fibrosis: 24% Stiffness: 11% Edema: 4% 2°: Fibrosis: 11% Stiffness: 11% Edema: 4%
IMRT VMAT IGRT	N = 2 pts^*^	1 day (median)	10.7	87.5%	0%	N/A	N/A	N/A	Major WC: 18.8% Minor WC: 12.5%	≥3°: 0%
3D-CRT	AI	1-2 weeks	5.9 yrs	82% 3 yrs 67% 6 yrs	11% 3 yrs 17% 6 yrs	N/A	N/A	N/A	Toxicity recorded for 17 pts: Acute WC: seromas/hematomas: 6 surgical site infection: 5 delayed wound healing: 1	

Author	Year and country	Type of trial and Inclusion criteria	N	Median age (years)	Sex ratio (♀:♂ in %)	Histologic grade	Location	Median tumor dia- meter	Fraction and dose; target volume	EQD2/BEDα/β of 4 (Gy)
Kubicek et al. [61]	2018 USA	Phase II single center trial Localized extremity STS	13	N/A, all patients > 18 yrs	N/A	G1-G2: 21.4% G3: 78.5%	LE: 71.4% UE: 14.3% Groin: 14.3%	7.6 cm	Most pts: 5 x 7 Gy = 35 Gy 3 of 13 pts: 5 x 8 Gy = 40 Gy Median isodose line: 81% CTV = GTV + 0.5 cm radial; + 3 cm long. PTV= CTV + 0.5 cm	Most pts: 64.17 Gy/96.3 Gy 3 of 13 pts: 80 Gy/120 Gy
Kılıç et al. [62]	2017 Turkey	Retrosp. Analysis Localized, G2-G3 ≥ 4cm or G1 ≥ 8cm extremity STS	67	47	43 : 57	G2: 7.5% G3: 26.9 % Unknown: 65.6%	N/A	9.6 cm	8 x 3.5 Gy = 28 Gy PTV: N/A	35 Gy/52.5 Gy
Koseła-Paterczyk et al. [63]	2016 Poland	Sub-analysis of (64)	32	50	41 : 59	Myxoid liposarcoma only G1: 15.6 % G2: 12.5 % G3: 46.9 % Unknown: 25%	LE: 97% UE: 3%	10.5 cm	53%: 5 x 5 Gy = 25 Gy 47%: 5 x 4 Gy = 20 Gy CTV = GTV + 2cm transv.; + 4cm long. PTV = CTV + 0.7-1cm	53%: 37.5 Gy/ 56.3 Gy 47%: 26.67 Gy/40 Gy
Koseła-Paterczyk et al. [64]	2014 Poland	Phase II single center trial Locally advanced trunk wall or extremity G2-G3 STS or G1 if > 10cm diameter	272	55	53 : 47	G1: 11.8 % G2: 23.6 % G3: 64.6 %	LE: 70.2% UE: 16.2% Trunk: 13.6%	8.5 cm	5 x 5 Gy = 25 Gy CTV = GTV + 2cm transv.; + 4cm long. PTV = CTV + 0.7-1cm	37.5 Gy/ 56.3 Gy

RT modality	CTX	Time to surgery	Median FU (months)	OS	LR	LC	LRFS	DFS	Acute toxicity	Late toxicity
SBRT	21.4%; agent N/A	37 days (median)	279 days	N/A	7.7%	93%	N/A	N/A	Major WC: 28.5 %	0%
N/A	50% RT+AI vs. 50% RT alone	2-3 Weeks after pre-op RT	37	3yrs: 74.1% vs. 90.0% p=0.4	14.9%	N/A	3 yrs: 77.1% vs. 76.3% p=0.86	3 yrs: 50.5% vs. 65.7% p=0.33	N/A	N/A
3D-CRT	No	3-7 days	60	68% 5 yrs	9.3%	90%^#^ 5 yrs	N/A^#^	N/A	Acute:22 % = 7 patients 3 wound infection 2 wound dehiscence 5 prolonged healing Late: 9% = 3 patients 1 prolonged edema 2 tissue fibrosis No differences in RT regimens	
3D-CRT IMRT for trunk lesions only (13.6%)	22.4% substance N/A	3-7 days	35	72% 3 yrs	19.1%	81% 3yrs**	N/A**	N/A	Overall: 32.4%: Inflammation requiring antibiotic treatment: 11.8% Wound dehiscence: 11.8% Prolonged wound healing: 16.5%	Overall: 14.7% 1°-3° Fibrosis: 3.7% Edema: 9.2%

The trial characteristics, patient characteristics, radiotherapy, chemotherapy, time to surgery as well as outcome parameters and rates for acute and late toxicity are included. 1° (grade 1), 2° (grade 2), 3° (grade 3), 3D-CRT (3D conformal radiotherapy), AI (doxorubicin/ifosfamide), BED (Biologically Effective Dose), cm (centimeter), CTV (clinical target volume), CTX (chemotherapy), DFS (disease-free survival), doxo (doxorubicine), DTIC (dacarbazine), EQD2 (Equivalent Dose in 2 Gy Fractions), FU (follow-up), G (grade), GTV (gross tumor volume), Gy (gray), IGRT (image-guided radiotherapy), IMRT (intensity modulated radiotherapy), LC (local control), LE (lower extremity), long. (longitudinally), (LR (local recurrence), LRFS (local recurrence-free survival), mths (months), N/A (not available), OS (overall survival), pts (patients), PTV (planning target volume) Retrosp. Analysis (retrospective analysis), RT (radiotherapy), SBRT (stereotactic body radiotherapy), STS (soft tissue sarcoma), transv (transversally), UE (upper extremity), USA (United States of America), VMAT (Volumetric Intensity Modulated Arc Therapy), WC (wound complication), yrs (years)

*One patient received gemcitabine/docetaxel 6 weeks post-op; one patient received doxorubicin/temsirolimus 1 week prior to RT

^#^The definition of LRFS included the events local recurrence or death. Notably, the trial applied the same definition, however, the value of 90% for 5-year LRFS is higher than the 5-year OS of 68%, which counters the definition of LRFS. The 5-year 90% value is therefore equivalent to the 5-year local control

**Death was not included as an event for the LRFS. The 81% is therefore equivalent to the 3-year LC rate

**Table 3:** The table summarizes major published studies on preoperative conventionally fractionated RT

Author	Year & country	Type of trial & inclusion criteria	N	Median age (years)	Sex ratio (♀:♂)	Histological grade	Location	Median tumor diameter	Fraction & dose; target volume	EQD2/α/β of 4 Gy	RT modality	CTX	Time to surgery	Median FU (months)	OS	LR	LC	LRFS	DFS	Acute toxicity	Late toxicity
Lansu et al. [65]	2021 Nether-lands	Phase II multi-center trial Localized extremity or trunk MLPS	79	45	44: 56	N/A	LE: 91% UE: 3% Trunk: 6%	9.9 cm	18 × 2 Gy = 36 Gy CTV = GTV + 3 cm long.; + 1.5 cm all other directions PTV = CTV + 1 cm	36 Gy/54 Gy	IMRT	No	≥ 4 weeks	25	95% 3 yrs	N/A	100%	N/A	N/A	Overall WC; 22%	2°: 11% 3°: 3%
Lansu et al. [66]	2019 Nether-lands	Retrosp. analysis Localized extremity STS	191	60	♀ n = 88 ♂ n = 103	G1: n = 14 G2: n = 76 G3: n = 79 N/A: n = 22	LE: 92% UE: 8%	N/A	25 × 2 = 50 Gy (85% of pts) CTV = GTV + 4 cm long.; + 1.5 cm all other directions PTV = CTV + 1 cm	50 Gy/75 Gy	EBRT	No	6 weeks (median)	21	70% 5 yrs	5%	93% 5 yrs	N/A	N/A	Overall WC: 31%	N/A
Wang et al. [35]	2015 USA	Phase II multi-center trial Localized extremity STS, < 32 cm	86	61	53: 47	G1: 16.5% G2: 26.6% G3: 48.1%	LE: 78.5% UE: 13.9% Other: 7.6%	10.5 cm	25 × 2 Gy = 50 Gy PTV = CTV + 0.5 cm ≥ 8 cm diameter or G2/G3: CTV = GTV + 3 cm long.; + 1.5 cm radial < 8 cm diameter or G1 CTV = GTV + 2 cm long., 1 cm radial	50 Gy/75 Gy	IG-IMRT 74.7% 3DCRT 25.3%	No	4–8 weeks	3.6 yrs	80.6% 2 yrs	N/A	94% 2 yrs	N/A	N/A	Major WC: 36.6%	≥ 2°: 10.5% at 2 yrs
O’Sullivan et al. [67]	2013 Canada	Phase II single center trial Localized lower extremity STS	59	56 (mean)	♀n = 29 ♂n = 30	G1: n = 4 G2: n = 26 G3: n = 29	LE only	9.5 cm	25 × 2 Gy = 50 Gy CTV = GTV + 4 cm long. + 1.5 cm radial PTV = CTV + 0.5 cm	50 Gy/75 Gy	IG-IMRT	No	N/A	49	N/A	6.8%	N/A	88.2% 5 yrs	N/A	Major WC: 30.5%	No > 2° toxicity
Hui et al. [68]	2006 Australia	Retrosp. analysis Localized extremity or trunk wall STS	67	52	♀n = 26 ♂n = 41	G1: n = 19 G2/G3: n = 46 N/A: n = 2	LE: n = 53 UE: n = 9 Trunk: n = 5	6 cm	28 × 1.8 Gy = 50.4 Gy PTV = GTV + 6 cm long.	48.72 Gy/73.08 Gy	EBRT	n = 3 pts: doxo post op	33 days (median)	4.1 yrs	73% 5 yrs	9%	N/A	93% 5 yrs	N/A	3°: Dermatitis: 6% overall WC: 41%	Overall: n = 5 pts
Kraybill et al. [69]	2006 USA	Phase II multi-center trial G2–G3 extremity and trunk wall sarcoma, ≥ 8 cm, ≤ 4 lung metastases	64	45.5	44: 56	G2: 20% G3: 80%	Extremity 88% Torso: 12%	15 cm	22 × 2 Gy interdigitated = 44 Gy PTV = GTV + 9 cm long. + ≥ 2 cm radial	44 Gy/66 Gy	EBRT	MAID	80 days after day 1 of CTX	6.1 yrs	75.1% 3 yrs	17.6% 3 yrs	N/A	N/A	56.6% 3 yrs	3°: Hematological: 13% Nonhematological: 28% 4°: Overall: 84% 5°: Overall: 5% Late toxicity: N/A	
Zagars et al. [70]	2003 USA	Retrosp. Analysis Localized, G1–G3 STS	271 pre op	N/A	N/A	G1: 4% G2: 26% G3: 70%	LE: 59% UE: 14% Other: 27%	8 cm	Median single dose: 2.0 Gy Median total dose: 50 Gy	50 Gy/75 Gy	EBRT	doxo	4–6 weeks	6.4 yrs	N/A	N/A	85% 5 yrs 83% 10 yrs	N/A	N/A	N/A	5%
O’ Sullivan et al. [25, 26] and Davis et al. [24]	2002 Canada	Phase III multi-center RCT Localized, extremity STS	94 pre op	< 50: 34% ≥ 50– < 70: 43% ≥ 70: 23%	45: 55	G1: 17% G2–G3: 83%	LE: 80% UE: 20%	≤ 10 cm 65% > 10 cm 35%	Pre op: 25 × 2 Gy = 50 Gy (n = 88 pts) Additional post op boost 8 × 2 Gy = 16 Gy (n = 14 pts) pre op PTV = GTV + 5 cm long. Post op boost: PTV = GTV + 2 cm	Pre op: 50 Gy/75 Gy post op boost: 16 Gy/24 Gy	EBRT	No	3–6 weeks	3.3 yrs	73%	N/A	93% 5 yrs	58% 5 yrs	N/A	Major WC: 35%	≥ 2°: Fibrosis: 31.5% Joint stiffness: 17.8% Edema: 15.1%
Pollack et al. [71]	1998 USA	Retrosp. analysis G2–G3 pleom. sarcoma, liposarcoma, synovial sarcoma	128 pre op	54 (mean)	58: 42	G2: 32.8% G3: 67.2%	LE + UE: 82% Other: 18%	10 cm (mean)	25 × 2 Gy = 50 Gy PTV = GTV + 5–7 cm long. + 2–3 cm radial	50 Gy/75 Gy	3D-CRT	doxo, CP, DTIC, VCR	N/A	97	N/A	N/A	82% 5 yrs	N/A	N/A	Acute WC: 25% (pre op)	6.2% (entire cohort)

The trial characteristics, patient characteristics, radiotherapy, chemotherapy, time to surgery as well as outcome parameters and rates for acute and late toxicity are included. 1° (grade 1), 2° (grade 2), 3° (grade 3), 3D-CRT (3D conformal radiotherapy), BED (Biologically Effective Dose), cm (centimeter), CP (cyclophosphamide) CTV (clinical target volume), CTX (chemotherapy), DFS (disease-free survival), doxo (doxorubicine), DTIC (dacarbazine), EBRT (external beam radiotherapy), EQD2 (Equivalent Dose in 2 Gy Fractions), FU (follow-up), G (grade), GTV (gross tumor volume), Gy (gray), IMRT (intensity modulated radiotherapy), IG-IMRT (Image-guided intensity-modulated radiotherapy), LC (local control), LE (lower extremity), long. (longitudinally), LR (local recurrence), LRFS (local recurrence-free survival), MLPS (myxoid liposarcoma), mths (months), N/A (not available), OS (overall survival), pts (patients),  pre op (preoperative), post op (postoperative), PTV (planning target volume), RCT (randomized controlled trial), Retrosp. analysis (retrospective analysis), RT (radiotherapy), STS (soft tissue sarcoma), transv (transversally), UE (upper extremity), USA (United States of America), VCR (vincristine), VMAT (Volumetric Intensity Modulated Arc Therapy), WC (wound complication), yrs (years)

## Discussion

Herein, we review the current literature on preoperative HFRT in the management of STS. The most frequently voiced criticism of this treatment approach concerns the following points: (i) the possibilty of increased toxicity with pre- and postoperative complications; (ii) assumed worse oncological outcomes compared to standard fractionated RT; (iii) financial concerns due to the reduced number of therapy sessions in HFRT [72, 73]. From a logistical and health economic standpoint, HFRT is undoubtedly the preferred and better applicable treatment modality for all patients and age groups seeking care at sarcoma centers [41, 42, 44]. Regional hyperthermia has historically been used in combination with chemotherapy showing promising results for the treatment of STS [74–78]. Combined with neoadjuvant chemotherapy, regional hyperthermia improves OS and local progression-free survival for patients with localized high-grade STS [79, 80]. As part of a first study, hypofractionated radiotherapy was combined with hyperthermia on 30 patients with marginally or unresectable, mostly G1 STS. This phase II feasibility study from the Warsaw sarcoma center by Spałek et al. met its primary endpoint of testing feasibility as it was well tolerated and adherence to the therapy protocol was successful [81]. Due to the scope of the present review to describe and compare preoperative HFRT to current standard treatment (normo-fractionated RT), trials on regional hyperthermia were not included.

### Acute and late toxicity

The first and foremost concern about increased early and late toxicity with HFRT cannot be confirmed based on the available data. Firstly, to define major WCs, most trials adopted their definition from the largest phase III trial (SR-2 trial) that compared toxicity rates in pre- vs. postoperative normofractionated RT. In this trial, a major WC was defined as a second surgery under general or regional anesthesia for wound repair up to four months after primary surgery. Additionally, aspiration of seromas, re-admission for wound care such as intravenous antibiotics or persistent deep packing for 120 days or beyond were included in that definition [26]. Preoperative RT was associated with a WC rate of 35%, while 17% of participants showed postoperative WCs (Table 3) [26].

In a 2021 published, non-controlled, interventional trial by Koseła-Paterczyk et al., 311 patients treated with a short preoperative course of 5 × 5 Gy showed lower WC rates of 28% compared to the SR-2 trial [53]. The average tumor size was even larger while the histological grade, tumor location, and median age of participants were comparable. Treatment planning was also similar in both trials: In the trial by Koseła-Paterczyk et al. the clinical target volume (CTV) was 2 cm transversally and 4 cm longitudinally. The planning target volume (PTV) was 1 cm in all directions (Table 2). In the SR-2 trial, preoperative RT treatment consisted of 25 × 2 Gy to a volume of 5 cm proximal and distal to the tissue at risk displayed on computed tomography (CT). A minor subgroup of patients with positive surgical margins after preoperative RT received a sequential boost (16–20 Gy in 2 Gy fractions) defined as lesion volume plus 2 cm in all directions.

Possible explanations for the difference in WC rates between both trials may be: (i) Increased precision by image-guided radiotherapy (IGRT) conducted via daily cone-beam CTs in the trial by Koseła-Paterczyk et al.; (ii) the use of contrast enhanced magnetic resonance imaging (MRI) fused with CT for planning, although the exact proportion of patients where MRI was applied is not given; (iii) a possible difference in the tumor depth as another risk factor for WC, also not given in the trial by Koseła-Paterczyk et al.; (iv) a difference in patients comorbidities (e.g. increased body mass index (BMI), smoking, diabetes) adversely affecting wound complication rates [82–85].

One essential limitation of the 2021 trial of Koseła-Paterczyk et al. is the absence of intensity modulated radiotherapy (IMRT) technique. It would have been interesting to observe whether adding IMRT techniques to the hypofractionated 5 × 5 Gy regimen would have reduced toxicity rates even more. In 2014, Koseła-Paterczyk et al. had applied HFRT to a comparable group of 272 patients (mostly G3 sarcomas located in the lower extremity), but without IMRT or IGRT. Herein, major WC rates were higher and similar to the rates in the SR-2 trial (32.4% vs. 35% in the SR-2), while late toxicities were less common, suggesting IMRT and IGRT as important influence parameters [64].

For normofractionated RT, more data exists suggesting a clear benefit of image-guided and intensity modulated radiotherapy (IG-IMRT) techniques. The group of O'Sullivan et al. published another trial showing beneficial toxicity rates by using IG-IMRT and standard target volume delineations [67]. Although the rate of WCs was numerically lower, yet not statistically significant, the need for tissue transfer was significantly reduced [67]. Supporting this approach, Wang et al. investigated the impact of normofractionated IGRT on toxicity rates in preoperative normofractionated RT for STS applying the same definitions for late toxicity and acute WCs as in the SR-2 trial [24, 26]. By adding IGRT, the late toxicity rate again dropped substantially to 10.5% in the RTOG-0630 trial [35].

Interestingly, two interventional trials evaluating stereotactic body radiotherapy (SBRT) used even higher doses of 5 × 8 and 5 × 7 Gy and revealed acute WC rates similar to conventional HFRT yet lower than in the normofractionated SR-2 trial (28% and 28.5% respectively) [55, 61]. Notable other adverse events were vascular occlusions described in a small proportion of patients after 5 × 8 Gy SBRT requiring disarticulation surgery (n = 3) and one case of amputation [55]. The amount of literature describing damage to tumor vasculature under intense hypofractionation has been growing recently [86, 87]. This effect has first been described in in vitro experiments after single fractions ≥ 10 Gy which may explain the described adverse effects [88]. Nevertheless, the SBRT data on STS are limited by the small number of participants (25 in the trial of Leite et al. vs. 13 in the trial of Kubicek et al.) and the short median follow-up of 9.3 months in the latter trial, which therefore could detect no late toxicities [55, 61]. Nevertheless, it is undoubted that advances in RT planning and techniques such as IGRT and IMRT have improved precision and reduced toxicity rates for STS patients. An upcoming Russian trial is currently recruiting patients for a 3-step sequence of preoperative stereotactic RT (5 × 5 Gy), surgery, and postoperative normofractionated RT (25 × 2 Gy). The primary endpoint is the complication rate after each step of the protocol [89](NCT04330456).

To further elucidate the effect of preoperative HFRT and chemotherapy on R0 limb-sparing surgery and toxicity rates for marginally resectable STS, a phase II trial with 46 patients from the Warsaw sarcoma center by Spałek et al. was published in 2021. R0 resection was achieved in 72% of patients while acute WCs were observed in 34% of patients comparable to the 35% in the SR-2 trial. Data on late toxicity rates are still pending [26, 54]. However, in this trial the median tumor diameter of 17.4 cm was remarkably larger compared to most other trials with perioperative HFRT for STS and to the SR-2 trial (< 10 cm in 65% in the preoperative RT group). Supporting this association, the multivariable analysis in the SR-2 trial also revealed a significant correlation between baseline tumor size and WCs [26]. Thus, having almost equal WC rates in hypofractionated and normofractionated RT despite a substantial difference in size attenuates the argument of increased WCs in HFRT for STS.

Only one trial has shown slightly higher rates of acute WCs using HFRT (37.9% vs. 35% in SR-2) [63]. However, in this trial, the sample size was relatively small (n = 34) because only myxoid liposarcomas (MLPS) were included. Moreover, most patients were irradiated with conventional 3D conformal radiotherapy (3D-CRT) and a short time gap of 3–7 days between RT and surgery [63]. Besides, MLPS are known for their favorable prognosis and radiosensitivity [90, 91]. So, even if further trials on this rare malignant tumor would bring forth more evidence of increased toxicity with HFRT, one could still discuss a de-escalation concept due to their high radiosensitivity. The Dutch multicenter DOREMY trial has applied reduced preoperative normofractionated RT (18 × 2 Gy instead of 25 × 2 Gy standard dose) for MLPS patients in an attempt to deescalate radiation dose. The authors achieved remarkably low acute WCs of 17% when compared to the preoperative RT group in the SR-2 trial. However, while the definition of major WC as a clinical diagnosis is equal, the DOREMY trial defined acute WCs by 30 days after surgery while the SR-2 trial applied 120 days [92] (NCT02106312).

A lot of knowledge on risk factors for major WCs stems from large surgical and RT data analyses. As such, it is an interesting finding throughout all treatment modalities and trials investigated in this review that the vast majority of WCs are located in the lower extremities, accounting for substantial postoperative morbidity (Table 2). This observation has been confirmed in different multicenter data analyses [84, 85]. In addition, the authors also found influenceable risk factors like increased BMI and smoking to be associated with postoperative WCs [84, 85]. In line with this, further trials confirmed the above-mentioned risk factors and added diabetes, tumor size > 10 cm, vascular tumor infiltration, and proximity to the skin < 3 mm as further predictors of major WCs [82, 83]. These findings may alter the preoperative management (nutrition, smoking cessation, diabetes training, surgical technique) to optimize post-surgical outcomes in STS patients [82, 83].

Furthermore, while acute WCs constitute serious adverse events, they are usually curable by local treatment. In contrast, long-term analysis of the patients in the Canadian SR-2 trials has revealed significantly lower functional scores in patients suffering from late and irreversible toxicities such as fibrosis, joint stiffness, and edema [24]. This observation may explain the increasing trend towards preferring pre- over postoperative RT in the treatment of STS [22, 23].

Apart from one trial, no other trials analyzed in our systematic review have found higher rates of early or late toxicity with HFRT for STS [63]. Quite the contrary, most trials have shown reduced risks of toxicity with advanced RT techniques. However, no large randomized phase III controlled trial has yet compared HFRT to normofractionated RT with a particular focus on toxicity rates and morbidity. One of the few controlled trials investigating this very topic is currently enrolling patients at the University of Wisconsin Hospital and Clinics (Madison, Wisconsin, United States, section 4.3 Upcoming data) [93].

### Oncological outcomes

The outcome benefits of HFRT for STS are promising. Well-established independent risk factors for LR and mortality comprise positive surgical margins, histological grade, tumor depth, and previous LR for subsequent recurrences and mortality. Additionally, specific histological subtypes (e.g., malignant peripheral nerve sheath tumor or myxofibrosarcomas) are associated with disadvantageous clinical outcomes [11, 12, 15, 16].

Overall, LC as a quality criterion for HFRT shows good to excellent results, ranging between 80–100% between 3 to 5 years in the largest studies analyzed herein (Table 2). The most comprehensive trial comprising 311 representative patients with locally advanced sarcomas treated with a short course of 5 × 5 Gy has achieved acceptable rates of 5-year LR of 13.8% when compared to previous literature [14, 53, 94]. About 83% of tumors were resected with clear margins, a protective factor for LR as described in previously published analyses [95]. The additional preoperative chemotherapy with doxorubicin and ifosfamide or dacarbazine administered to one third of patients did not significantly alter survival or LR, although the trial was not powered for this factor [53]. On multivariable analysis, specific histological subtypes such as malignant peripheral nerve sheath tumors or leiomyosarcomas have confirmed the previous literature on their increased malignancy and resistance to treatment (5-year LC of approximately 65–70%) [11, 96].

Again, the addition of IG-IMRT to HFRT has substantial benefits and improves LC rates. Kalbasi et al. have applied 5 × 6 Gy IMRT in 76% of patients and IGRT in almost all 50 patients enrolled in 2020 [47]. With a minimum follow-up of two years, only 5.7% of patients with LR were observed [47]. Limitations in comparability are the pending long-term follow-up data [47]. The improvement by IMRT is supported by data on normofractionated postoperative RT, where IMRT has shown significant benefits on LC compared to conventional external beam RT [97, 98]. Altogether, the presented data on preoperative HFRT has shown similar LC rates when compared to preoperative normofractionated RT for STS [70, 71].

An interesting secondary finding in the study by Kalbasi et al. is the significant increase in both patient accrual and distance traveled by patients, when they were enrolled into 5 × 6 Gy RT compared to standard 25 × 2 Gy in the 2-year period preceding study initiation [47]. This approves the logistical and convenience argument by many other studies on patient preferences and therapy adherence to shorter RT courses, which particularly holds true for elderly patients [44, 46, 99].

MLPS repeatedly stand out by their remarkably high radiosensitivity, which sustains also in HFRT regimens. In 27 patients with large MLPS (median size: 13 cm), treated with preoperative 5 × 5 Gy and a median follow-up of 27 months, none of the patients had a LR. OS was 93% because of two patients who died after metastatic spread [58]. In another trial, published four years earlier, the same authors from the Warsaw sarcoma center have used 5 × 5 or 5 × 4 Gy for MLPS patients and have shown similarly favorable LC rates of 90% after five years. The 5-year OS was 68%. All deaths were related to distant recurrences, again proving the excellent radiosensitivity and local controllability by HFRT [63]. This radiosensitivity is confirmed in multiple previous studies and large database analyses on normofractionated RT and may be exploited to further deescalate local therapy regimens [90, 91, 100].

We can therefore conclude that the present data strongly suggests modern HFRT regimens and techniques to be comparable to normofractionated RT in LC rates of STS. However, the present results are, at best, derived from phase II trials. So far, no randomized phase III trial comparing normofractionated RT to HFRT for STS has been conducted. Both the study population and the specific tumor entities are highly heterogeneous, and most of the trials are non-controlled trials or retrospective data analyses (Table 2) [101]. The included articles demonstrated moderate to serious overall risk of bias and therefore hamper comparability (Additional file 2: Risk of bias assessment according to ROBINS-I, Table 1). Moreover, the available trials differ in RT, surgical techniques, concomitant chemotherapy regimens, and the therapy modalities’ order. Research on STS as "orphan diseases" is impeded by low prevalence and lower funding compared to other cancer entities [102]. Thus, the present data is generating strong hypotheses and future results are eagerly awaited.

### Upcoming data

More than 15 trials on HFRT + / − chemotherapy in STS are currently ongoing (Table 4). Due to the low prevalence, most trials have long recruiting phases. Among the first trials to compare conventionally fractionated vs. HFRT for STS has recently begun accruing patients at the University of Wisconsin, USA [93] (NCT05109494). Another randomized interventional trial focuses on acute postoperative WCs in localized head and neck, trunk and extremity STS after 14 × 3 Gy preoperative RT (study arm B) compared to standard preoperative RT (25 × 2 Gy) [103]. The study began recruiting in June 2021 at two Dutch university medical centers in Leiden and Groningen and is expected to reach primary completion by April 2025 [103] (NCT04425967).

**Table 4:** The table summarizes currently ongoing and recruiting trials on preoperative hypofractionated radiotherapy for soft tissue sarcoma

NCT number/phase	Title	RTX fraction × dose	Outcome measures	Dates	Center
NCT05109494/Phase II	Hypofractionated vs Conventional Fractionated RT in Soft Tissue Sarcomas	25 × 2 Gy = 50 Gy vs 5 × 5.5 Gy = 27.5 Gy	1°: Pathological necrosis 2°: Surgical margins, WC, late toxicity, PFS, LR	Start: December 2021 Study completion: November 2026	University of Wisconsin Hospital and Clinics, Madison, Wisconsin, United States
NCT04425967/Phase II	Short Course Of Preoperative Radiotherapy in Head and Neck-, Trunk- and Extremity Soft Tissue Sarcomas	25 × 2 Gy = 50 Gy vs 14 × 3 = 42 Gy	1°: Acute toxicity (30 days post op) 2°: LC, late toxicity (2 years)	Start: June 2021 Study completion: April 2034	Universitair Medisch Centrum Groningen, Groningen, Netherlands Leids Universitair Medisch Centrum, Leiden, Netherlands Radboudumc, Nijmegen, Netherlands
NCT04562480/Phase II	Hypofractionated Radiation Therapy Before Surgery for the Treatment of Localized, Resectable Soft Tissue Sarcoma of the Extremity and Superficial Trunk	15 × 2.85 Gy = 42.75 Gy	1°: Major WC (within 120 days) 2°: LR, DFS, OS, late toxicity, pattern of relapse, QoL changes	Start: November 2020 Study completion: November 2026	Mayo Clinic in Rochester, Rochester, Minnesota, United States
NCT03819985/Phase II	Shorter Course, Hypofractionated Pre-Surgery Radiation Therapy in Treating Patients With Localized, Resectable Soft Tissue Sarcoma of the Extremity of Superficial Trunk	15 × 2.85 Gy = 42.75 Gy vs 25 × 2 Gy = 50 Gy (historical control)	1°: Non-inferiority design for time till major WC (within 120 days) 2°: LRFS, DFS, Time to relapse, Disease specific survival time, pattern of local relapse, acute toxicity other than WC, late toxicity, functional outcomes, QoL	Start: December 2018 Study completion: August 2023	MD Anderson Cancer Center, Houston, Texas, United States
NCT04617327/Phase I/II	Pre-operative RadiothErapy for Soft Tissue SarcOmas (PRESTO)	5 × 7 Gy = 35 Gy every other day (3 fractions per week)	1°: Acute toxicity (within 1 month) according to CTCAE V.5 2°: Performance measure by Physicians Muscle Tumor Rating Scale	Start: June 2020 Study completion: December 2027	McGill University Health Centre-Cedars Cancer Centre, Montréal, Quebec, Canada
NCT03972930/Phase II	Hypofractionated Radiotherapy for Soft Tissue Sarcomas	Highly conformal RT in 3–8 fractions maximum prescribed dose, total of 60 Gy in ≤ 8 weeks	1°: LC (2-year) 2°: LC (5-year), CR-rate, PFS, OS, acute toxicity, late toxicity	Start: June 2019 Study completion: July 2026	University of Wisconsin, Madison, Wisconsin, United States
NCT04946357/Phase II	Neoadjuvant Irradiation of Extremity Soft Tissue Sarcoma With Ions (EXTREM ION)	Proton: 13 × 3 Gy = 39 Gy (RBE) vs Carbon ion: 13 × 3 Gy = 39 Gy (RBE)	1°: Absence of wound healing disorders (till 120 days after surgery) 2°: LC, LPFS, DFS, OS	Start: June 2021 Study completion: July 2023	University Hospital Heidelberg, Heidelberg, Germany
NCT02634710/Phase II	Hypofractionated Pre-operative Radiation Therapy for Soft Tissue Sarcomas of the Extremity and Chest-wall	5 × 7 Gy = 35 Gy every other day	1°: LC (2 year) 2°: Serious adverse events (CTCAE V.4.0), Musculoskeletal Tumor Rating Scale Score, QoL, DFS, OS, radiological changes (T2 MRI), pathological changes	Start: February 2016 Study completion: December 2025	Froedtert Hospital, Milwaukee, Wisconsin, United States

The National Clinical Trial number, the study phase, study title, radiotherapy fractionation and dose, the primary and secondary outcomes, the dates and the participating centers are included. 1° (primary), 2° (secondary), CR (complete remission), CTCAE (Common Terminology Criteria of Adverse Events), DFS (disease-free survival), doxo (doxorubicine), LC (local control, LPFS (local progression-free survival), LR (local recurrence), LRFS (local recurrence-free survival), MRI ( Magnetic resonance imaging), NCT (National Clinical Trial), OS (overall survival), QoL (quality of life), RBE (relative biological effectiveness), WC (wound complication)

Many studies are testing different preoperative, HFRT regimens to shorten therapy time and improve patient convenience. For instance, 15 × 2.85 Gy is applied to investigate major WCs (as defined by O'Sullivan et al.) for an estimated number of 120 STS patients at the Mayo Clinic, Rochester, USA [26]. Secondary outcome measures include oncological outcomes and for the first time, patient reported outcomes with regard to changes in the quality of life. Estimated primary completion is November 2025 [104] (NCT04562480). The same regimen also investigating major WC rates in localized, resectable STS and comparing them to historical controls is conducted at the M.D. Anderson Cancer Center and expected to reach completion by August 2023 [105] (NCT03819985). Similarly, the McGill University in Montreal, Canada, is accruing patients to apply a short, preoperative, HFRT regimen of 5 × 7 Gy within one week (PRESTO trial). The primary outcome is radiation-associated toxicity. For the secondary outcomes, the authors apply established questionnaires and functional scoring systems (Toronto Extremity Salvage Score [TESS], Musculoskeletal Tumor Society Score MSTS) to evaluate patients' daily performance activity and quality of life. The study commenced in June 2020 and is estimated to reach primary completion by January 2025 [106] (NCT04617327).

Other groups apply evolving technology to improve outcomes for STS patients under HFRT: Another phase II trial at the University of Wisconsin will be accruing around 48 patients to test advanced highly conformal HFRT with 2-year LC rates as primary endpoint; the estimated primary completion date is July 2023 [107] (NCT03972930). Moreover, two phase II randomized German trials are investigating the feasibility of modern, neoadjuvant, hypofractionated particle therapy (C12 carbon ions vs. protons) with 3 Gy to 39 Gy for STS of the extremities and retroperitoneal STS. Both are currently accruing patients at the University of Heidelberg [108, 109] (NCT04946357 and NCT04219202).

## Summary

STS are rare, heterogenous malignancies and therefore challenging in both research and multidisciplinary treatment. Preoperative, five to six weeks RT regimens currently represent the mainstay of management at high-volume sarcoma centers in high-grade STS (G2-G3). Shortening RT courses can improve therapy convenience, raise cost-effectiveness, and provide more treatment opportunities for a wider range of patients. The suggested risk of higher rates of adverse effects and worse oncological outcomes cannot be confirmed by the available data and studies. Toxicity rates are mostly equal or less than in representative trials for normofractionated RT. Preoperative RT is preferred over postoperative RT due to lower rates of irreversible late toxicity. Preoperative HFRT achieves comparable LC rates with shorter duration of therapy. However, all data are derived from retrospective data analyses and phase II trials. The interpretation must therefore be made with caution. Multiple trials on HFRT are underway and the results in this evolving field are awaited with great interest.

## Supplementary Information

**Additional file 1**. The PRISMA 2020 checklist.

**Additional file 2**. Risk of bias assessment according to ROBINSI.

## Data Availability

Not applicable.

## Abbreviations

3D-CRT: 3D conformal radiotherapy
AI: Doxorubicin/ifosfamide
BMI: Body mass index
CT: Computed tomography
CTV: Clinical target volume
CTX: Chemotherapy
DFS: Disease-free survival
GTV: Gross tumor volume
HFRT: Hypofractionated radiotherapy
IGRT: Image-guided radiotherapy
IG-IMRT: Image-guided intensity modulated radiotherapy
IMRT: Intensity modulated radiotherapy
LC: Local control
LR: Local recurrence
LRFS: Local recurrence-free survival
MLPS: Myxoid liposarcomas
MRI: Magnetic resonance imaging
MSTS: Musculoskeletal Tumor Society Score
OS: Overall survival
PFS: Progression-free survival
PRISMA: Preferred Reporting Items for Systematic Reviews and Meta-Analyses
PTV: Planning target volume
ROBINS-I: Risk of Bias In Non-randomized Studies of Interventions tool
RT: Radiotherapy
SBRT: Stereotactic body radiotherapy
STS: Soft tissue sarcoma
TESS: Toronto Extremity Salvage Score
USA: United States of America
VMAT: Volumetric Intensity Modulated Arc Therapy
WC: Wound complication
