# Supplementary material for: Preoperative hypofractionated radiotherapy for soft tissue sarcomas: a systematic review
Source: Radiat Oncol. 2022 Sep 14;17:159. doi: 10.1186/s13014-022-02072-9 (PMC9472188; doi:10.1186/s13014-022-02072-9)
Supplement: Supplementary file 2 — Additional file 2. Risk of bias assessment according to ROBINSI. [file 13014_2022_2072_MOESM2_ESM.docx]

| Supplementary table 1: Risk of bias assessment according to the ROBINS-I tool (51). | | | | | | | | |
| --- | --- | --- | --- | --- | --- | --- | --- | --- |
| **Reference** | **Confounding  bias** | **Selection**  **bias** | **Classification of**  **intervention bias** | **Deviation from intended intervention bias** | **Missing data**  **bias** | **Measurement of outcome bias** | **Selection of reported results bias** | **Overall risk of bias** |
| Koseła-Paterczyk et al. (53) | Moderate | Low | Low | Low | Low | Low | Low | Moderate |
| Spalek et al. (54) | Moderate | Low | Low | Low | Low | Low | Low | Moderate |
| Leite et al. (55) | Moderate | Low | Low | Moderate | Moderate | Moderate | Low | Moderate |
| Potkrajcic et al. (56) | Serious | Low | Moderate | Low | Low | Moderate | Low | Serious |
| Silva et al. (57) | Moderate | Low | Low | Moderate | Low | Moderate | Low | Moderate |
| Koseła-Paterczyk et al. (58) | Moderate | Low | Low | Low | Low | Moderate | Low | Moderate |
| Kalbasi  et al. (47) | Moderate | Low | Low | No information | Moderate | Moderate | Low | Moderate |
| Parsai  et al. (59) | Serious | Low | Moderate | Low | Low | Low | Low | Serious |
| Pennington et al.  (60) | Moderate | Low | Serious | Moderate | Moderate | Low | Moderate | Serious |
| Kubicek et al.  (61) | Serious | Low | Low | Serious | Low | Moderate | Low | Serious |
| Kılıç et al. (62) | Moderate | Low | Moderate | Moderate | Low | Low | Low | Moderate |
| Koseła-Paterczyk et al. (63) | Serious | Low | Low | Low | Low | Moderate | Low | Serious |
| Koseła-Paterczyk et al. (64) | Moderate | Low | Low | Moderate | Low | Low | Moderate | Moderate |
